# Small-Cell Lung Cancer with Positive Anti-NMDAR and Anti-AMPAR Antibodies Paraneoplastic Limbic Encephalitis

**DOI:** 10.1155/2016/3263718

**Published:** 2016-12-13

**Authors:** Sabina Boangher, Pascal Mespouille, Corina-Mihaela Filip, Sophie Goffette

**Affiliations:** ^1^Department of Neurology, Arlon Hospital, No. 137, Rue des Déportés, 6700 Arlon, Belgium; ^2^Oncology Department, CH Luxembourg, Rue Nicolas-Ernest Barblé, No. 4, L 1210 Luxembourg, Luxembourg

## Abstract

We report the case of a 66-year-old woman, with paraneoplastic limbic encephalitis, treated 6 months earlier for bladder neoplasia. The patient presented to the emergency room with rapidly increasing symptoms, noninfectious cerebral spinal fluid associated with positive anti-NMDAR (as well as in serum) and positive AMPAR antibodies in the serum. Four months later, the patient was diagnosed with a small-cell lung cancer for which chemotherapy and radiotherapy was commenced. Simultaneously, endoscopic surgical treatment was undertaken for an in situ relapse of the bladder neoplasm. After the completion of 3 cycles of chemotherapy her neurological status temporarily worsened. The cerebral MRI did not show signs of encephalitis such as increased T2/FLAIR signal intensity in the mesial temporal lobes and limbic systems. No specific treatment was prescribed. Limbic encephalitis can be associated with malignant tumors such as lung carcinoma. Several cases reported in the literature have shown cognitive improvement after tumoral therapy. Regarding our experience, significant progress was achieved through immuno-modulatory treatment. A transitory deterioration of the cognitive process was perceived during the chemotherapy sessions.

## 1. Introduction

Limbic encephalitis (LE) as first described in 1967 [1] is characterized by an acute or subacute onset, memory loss, psychiatric features, and often seizures. Abnormal movements and autonomic instability had also been described. The neuropathological features consist in inflammation of the temporal lobe parenchyma and sometimes cortex edema. Several classes of antibodies (Abs) have been described in association with LE: the classical onconeuronal Abs directed against intracellular antigens and those directed against surface proteins (against N-methyl-D-aspartate [NMDA] receptor, *α*-amino-3-hydroxy-5-methyl-4-isoxazolepropionic acid receptor [AMPA], gamma aminobutyric acid (b) GABAb receptor, and the glycine receptor) and antibodies against neuronal cell adhesion molecules (leucine-rich glioma-inactivated 1 [LGI1] previously considered Abs against voltage-gated potassium channel (VGKC) and contactin-associated protein-like 2 [Caspr2]) [2].

## 2. Case Report

We describe the case of a 66-year-old female, diagnosed with in situ bladder carcinoma in December 2014. The patient's medical history included 1 episode of moderate depression and an autoimmune hepatitis, treated with cortisone.

After transurethral resection, she was treated with 40 mg Mitomycin C per intravesical instillation, one per week for 6 weeks and then one instillation per month for three months. No neurological side effects are commonly associated with Mitomycin intravesical administration.

In May 2015, 2 months after chemotherapy was completed the patient was admitted in the emergency room with confusion, short-term memory loss, anxiety, and aggressiveness in the absence of fever. The initial check-up excluded all organic causes, rendering the patient susceptible for a maniacal episode for which admission to the psychiatry proved reasonable.

A few days later a second check-up was made. The clinical exam was normal. The brain scan, as well as the EEG, showed no specific abnormalities. The analysis of cerebrospinal fluid (CSF) showed only a mild pleocytosis (18 white cells, predominant lymphocytes), a normal level of glucose and proteins. No intrathecal synthesis of immunoglobulin was described. Antibodies against NMDAR were positive both in serum and in CSF and Abs against AMPAR (type 2 subunit) were positive in the serum. The classical onconeuronal Abs and the other Abs directed against surface proteins were negative in the serum and CSF. The patient was then transferred to our neurological facility for treatment. She received intravenous methylprednisolone 1 g/day for 5 days and plasma exchange (5 exchanges in 10 days).

Afterwards we continued treatment with an immunosuppressant therapy, which could consist of either Rituximab alone or a combination of Cyclophosphamide and Rituximab. As case reports indicate, there is a decreased risk of recurrence with the prolonged use of immunosuppressive drugs such as Mycophenolate or Azathioprine [3]. In our case, we had a relative contraindication of the Cyclophosphamide because of the bladder carcinoma and, unfortunately, we did not have access to Rituximab. We chose Mycophenolate mofetil over Azathioprine based on our own medical practice because in this indication there is no data that supports one treatment over the other.

The patient's clinical and mental status improved at the end of acute treatment, but episodes of confusion and echolalia persisted nonetheless. Short-term memory loss symptoms started to fade away, in terms of remembering names of her family members, alongside with a more calm and decent behavior.

The cerebral MRI did not show increased signal intensity in the temporal lobes.

The CSF check-up was normal. The gynecological exam, the cystoscopy, the gastroscopy, and the colonoscopy were irrelevant. The thoracic-abdominal-pelvic scan showed no suspicion of any tumoral mass, although a nonspecific lymphadenopathy was reported in the right hilar zone. The positron emission tomography was tapering in the same area and therefore by means of a transesophageal ultrasound guided biopsy we managed to diagnose a small-cell lung carcinoma. The patient started in September 2015 4 cycles of chemotherapy with Carboplatin (AUC 5-6 IV on day 1) plus etoposide (100 mg/m^2^ IV on days 1–3 every 28 days) and concurrent radiotherapy (60 Gy/30 Fr) which yielded a complete response.

At the same time, she also presented an in situ relapse of her bladder neoplasm that required only an endoscopic surgical intervention.

After 3 sessions of chemotherapy the patient's neurological status deteriorated, hence leading to readmission. The cerebral MRI showed no signs of inflammation. The symptoms, the anxiety, and confusion spontaneously improved within 48 hours.

From October 2015 to July 2016 the patient was stable, but no improvement of her neurological deficit was witnessed since the treatment of the pulmonary neoplasm was started.

## 3. Discussion

Limbic encephalitis can be either paraneoplastic or a nonparaneoplastic [3, 4] autoimmune disorder associated with the presence of specific antibodies. The detection of neural-specific autoantibodies may confirm the diagnosis, predict immunotherapy response, and guide a cancer pursuit in paraneoplastic cases. Autoantibody testing may be performed on serum, cerebrospinal fluid, or both. In nonparaneoplastic cases, high serum levels of VGKC Abs [4] have been found, as well as Abs directed against GAD [5] or NMDA or to unknown antigens [6].

Some autoantibodies are strongly associated with malignancy, such as antibodies directed against the NMDA-receptor or the AMPA receptor.

The NMDAR antibodies, described for the first time in 2007 by Dalmau et al., are present in serum and in CSF, usually with intrathecal synthesis and are sometimes only detected in the CSF. The main target epitopes are in the NR1/NR2 heteromers of the NMDAR. The major antigen is an extracellular epitope of NR1/NR2 B subunit, which is predominantly expressed in the hippocampus and forebrain, but reactivity with other NR1/NR2 heteromers could also be observed [7].

Since 2007 anti-NMDA-receptor limbic encephalitis has become one of the most frequently recognized autoimmune encephalitides [8]. The disease predominates in women (81%) and young patients [9]. The presence of a tumor depends on age and gender. Young women have the highest chance of an underlying tumor (53%) [9]. The most frequently associated tumors are mature (dermoid cyst) or immature teratomas of the ovary in women and testicular germ-cell tumors in men [10], but other cancers like small-cell lung cancer (SCLC) [11] or breast, thymus, or pancreas cancers may be associated. These unusual tumors are more common in patients older than 45 [10]. Occasional cases of nonparaneoplastic anti-NMDA-receptor limbic encephalitis have also been described [6].

The AMPAR antibodies target extracellular epitopes of glutamate receptor type 1 or type 2 subunits, causing receptor cross-linking and internalization, thus resulting in a reversible decrease in AMPAR clusters at synapse level. The receptors are localized in the cerebral cortex, basal ganglia, and the cerebellum and they are implicated in learning and memorization mechanisms. In 70% of the cases a tumor is associated (thymus, breast, and lung) [12].

No case report of the simultaneous presence of the 2 Abs as described in our case was found in PubMed.

No data concerning the association between paraneoplastic encephalitis and bladder carcinoma has been found.

Brain MRI in patients with limbic encephalitis could be either normal or abnormal. The MRI abnormalities are usually found in the frontobasal and insular regions, but FLAIR signal hyperintensity was seen in the hippocampi, cerebellar or cerebral cortex, basal ganglia, and brainstem. Also, a case of anti-NMDA Abs encephalitis concomitant with multifocal subcortical white mater lesions was recently published [13].

The treatment of limbic encephalitis is controversial. There is no data supporting one treatment over another. Usually, patients are treated with a first-line immunotherapy including IV corticosteroids, IV immunoglobulins, and plasma exchange or with a combination of all these. The response to the treatment varies, depending on type of Abs. For the patients with anti-NMDA-receptors Abs, the response is influenced by the presence of a tumor. Those presenting with a tumor responded better to the first line of treatment regime than those without one. Even if long time outcome has not been established as being better, treating the tumor accelerates improvement and decreased relapses [14]. Patients without an associated tumor required a second-line or event line immunotherapy.

To the best of our knowledge this is the first case described of paraneoplastic limbic encephalitis which presents simultaneous two surface antibodies and two carcinomas.

## References

[1] Corsellis J. A. N., Goldberg G. J., Norton A. R. (1968). ‘Limbic encephalitis’ and its association with carcinoma. *Brain*.

[2] Lancaster E., Martinez-Hernandez E., Dalmau J. (2011). Encephalitis and antibodies to synaptic and neuronal cell surface proteins. *Neurology*.

[3] Dalmau J., Rosenfeld M. R. (2008). Paraneoplastic syndromes of the CNS. *The Lancet Neurology*.

[4] Vincent A., Buckley C., Schott J. M. (2004). Potassium channel antibody‐associated encephalopathy: a potentially immunotherapy‐responsive form of limbic encephalitis. *Brain*.

[5] Cianci V., Labate A., Lanza P. (2010). Non-paraneoplastic limbic encephalitis characterized by mesio-temporal seizures and extratemporal lesions: a case report. *Seizure*.

[6] Graus F., Saiz A., Lai M. (2008). Neuronal surface antigen antibodies in limbic encephalitis: clinical-immunologic associations. *Neurology*.

[7] Dalmau J., Tüzün E., Wu H.-Y. (2007). Paraneoplastic anti-*N*-methyl-D-aspartate receptor encephalitis associated with ovarian teratoma. *Annals of Neurology*.

[8] Granerod J., Ambrose H. E., Davies N. W. (2010). Causes of encephalitis and differences in their clinical presentations in England: a multicentre, population-based prospective study. *The Lancet Infectious Diseases*.

[9] Titulaer M. J., McCracken L., Gabilondo I. (2013). Treatment and prognostic factors for long-term outcome in patients with anti-NMDA receptor encephalitis: an observational cohort study. *The Lancet Neurology*.

[10] Leypoldt F., Wandinger K.-P., Bien C. G., Dalmau J. (2013). Autoimmune encephalitis. *European Neurological Review*.

[11] Qin Y.-Z., Chen Y.-Y., Bing Z.-X. (2013). Lung cancer associated paraneoplastic limbic encephalitis: an analysis of 7 cases. *Zhonghua wai ke za zhi [Chinese journal of surgery]*.

[12] Lai M., Hughes E. G., Peng X. (2009). AMPA receptor antibodies in limbic encephalitis alter synaptic receptor location. *Annals of Neurology*.

[13] Wang R.-J., Chen B.-D., Qi D. (2015). Anti-N-methyl-D-aspartate receptor encephalitis concomitant with multifocal subcortical white matter lesions on magnetic resonance imaging: a case report and review of the literature. *BMC Neurology*.

[14] Dalmau J., Gleichman A. J., Hughes E. G. (2008). Anti-NMDA-receptor encephalitis: case series and analysis of the effects of antibodies. *The Lancet Neurology*.

